# Correction: Sensory restoration by epidural stimulation of the lateral spinal cord in upper-limb amputees

**DOI:** 10.7554/eLife.72438

**Published:** 2021-07-26

**Authors:** Santosh Chandrasekaran, Ameya C Nanivadekar, Gina McKernan, Eric R Helm, Michael L Boninger, Jennifer Collinger, Robert Gaunt, Lee E Fisher

Chandrasekaran S, Nanivadekar AC, McKernan G, Helm ER, Boninger ML, Collinger JL, Gaunt RA, Fisher LE. 2020. Sensory restoration by epidural stimulation of the lateral spinal cord in upper-limb amputees. *eLife* **9**:e54349. doi: 10.7554/eLife.54349.

Published 21, July 2020

After publication, we became aware of a change in the software that controls our stimulator, which caused the polarity (anode vs. cathode) of stimulation pulses to be reversed for experiments with Subject 4. All stimulation parameters were within the safety limits outlined in our protocols that were approved by the University of Pittsburgh Institutional Review Board. Further, we do not believe that this change materially impacts any of the results reported in the paper. We have corrected the manuscript as follows to reflect this difference.

#1

Original text:

Figure 2 Dermatomal organization of the evoked percepts. (A) Schematic of dermatomes, adapted from Lee et al., 2008. Overlapping dermatome areas are shown in lighter shades. Dotted lines indicate our division of different regions of the fingers, hand, and arm. (B) An example of the segmentation of the spinal cord (from Subject 4) used to determine the location of each stimulation electrode. (C) Heat maps show the relative proportion of electrodes located at different spinal levels to the total number of percepts emanating from a specific region of the arm. The spinal level of each electrode was defined by the position of the cathode with respect to the spinal levels as seen in the X-rays. Spinal levels that have no electrodes nearby are marked with gray hatching.

Corrected text:

Figure 2 Dermatomal organization of the evoked percepts. (A) Schematic of dermatomes, adapted from Lee et al., 2008. Overlapping dermatome areas are shown in lighter shades. Dotted lines indicate our division of different regions of the fingers, hand, and arm. (B) An example of the segmentation of the spinal cord (from Subject 4) used to determine the location of each stimulation electrode. (C) Heat maps show the relative proportion of electrodes located at different spinal levels to the total number of percepts emanating from a specific region of the arm. For Subjects 1-3, the spinal level of each electrode was defined by the position of the cathode with respect to the spinal levels as seen in the X-rays. For Subject 4, the spinal level of each electrode was defined by the position of the anode. Spinal levels that have no electrodes nearby are marked with gray hatching.

#2

Original text:

Stimulation pulse trains were charge-balanced, cathodic-first square pulses, with either asymmetric or symmetric cathodic and anodic phases. For asymmetric pulses, the anodic phase was twice the duration and half the amplitude of the cathodic phase.

Corrected text:

Stimulation pulse trains were charge-balanced square pulses, with either asymmetric or symmetric cathodic and anodic phases. For Subjects 1-3, the first phase of stimulation was cathodic, while for Subject 4, an error in the stimulation control code caused the first phase of stimulation to be anodic. For asymmetric pulses, the second phase was twice the duration and half the amplitude of the first phase.

The article has been corrected accordingly.

