# Supplementary material for: Sensory restoration by epidural stimulation of the lateral spinal cord in upper-limb amputees
Source: eLife. 2020 Jul 21;9:e54349. doi: 10.7554/eLife.54349 (PMC7373432; doi:10.7554/eLife.54349)
Supplement: Supplementary file 2. — For detection and discrimination trials the threshold (TH) and JND per stimulation channel are listed along with the corresponding frequency and pulse width that were used. [file elife-54349-supp2.docx]

**Supplementary File 1:** Summary of psychophysics testing for each subject. For detection and discrimination trials the threshold (TH) and JND per stimulation channel are listed along with the corresponding frequency and pulse width that were used.

| **Subject** | **Electrode** | **Receptive Field** | **minimum modal amplitude (mA)** | **Detection** | | | **Discrimination** | | | | **Magnitude estimation** | | |
| --- | --- | --- | --- | --- | --- | --- | --- | --- | --- | --- | --- | --- | --- |
|  |  |  |  | **TH (mA)** | **F**  **(Hz)** | **PW (µs)** | **JND*_low_* (µA)** | **JND*_high_* (µA)** | **F**  **(Hz)** | **PW**  **(µs)** | **slope** | **R^2^** |  |
| 1 | 1 | D1–D2 | 5.48 | 3.75 | 100 | 200 |  | 618 | 100 | 1000 | 0.37 | 0.62 |  |
|  | 2 | Palm (ulnar) | 5.0 |  |  |  |  |  |  |  | 1.07 | 0.42 |  |
|  | 3 | Palm (ulnar) | 4.0 | 3.75 | 100 | 200 |  |  |  |  | 1.7 | 0.68 |  |
| 2 | 1 | Hand | 4.0 | 1.14 | 20 | 200 |  |  |  |  | 3.18 | 0.78 |  |
|  | 2 | Thumb | 3.0 | 1.21 | 20 | 200 | 245 |  | 20 | 200 | 2.36 | 0.71 |  |
|  | 3 | Hand | 4.0 |  |  |  |  |  |  |  | 2.21 | 0.76 |  |
|  | 4 | Palm, D1 | 3.0 | 1.11 | 20 | 200 |  |  |  |  | 2.74 | 0.7 |  |
|  | 5 | Thumb | 3.0 | 0.92 | 20 | 200 |  |  |  |  | 1.2 | 0.33 |  |
| 3 | 1 | Hand | 6.0 | 1.98 | 50 | 200 |  |  |  |  | 0.94 | 0.74 |  |
|  | 2 | Hand | 5.0 | 2.17 | 50 | 200 |  |  |  |  | 1.33 | 0.73 |  |
|  | 3 | Hand | 5.0 | 1.67 | 50 | 200 | 151 |  | 50 | 200 | 1.27 | 0.76 |  |
|  | 4 | Palm, D1–D4 | 3.0 | 0.97 | 50 | 200 |  |  |  |  |  |  |  |
|  | 5 | Palm, D3–D4 | 4.0 | 1.28 | 50 | 200 |  |  |  |  | 1.22 | 0.87 |  |
|  | 6 | Palm, D1–D4 | 5.0 | 1.53 | 50 | 200 |  |  |  |  | 1.18 | 0.74 |  |
|  | 7 | Palm, D1–D4 | 4.0 | 1.48 |  |  |  |  |  |  | 1.58 | 0.79 |  |
|  | 8 | Palm, D3–D4 | 5.0 | 1.65 | 50 | 200 |  |  |  |  | 1.39 | 0.81 |  |
|  | 9 | Palm, D2–D4 | 3.0 |  |  |  |  |  |  |  | 1.43 | 0.69 |  |
| 4 | 1 | Hand | 3.0 | 1.52 | 50 | 200 |  |  |  |  |  |  |  |
|  | 2 | D2, D4 | 4.0 |  |  |  |  |  |  |  |  |  |  |
|  | 3 | D2 | 2.0 | 1.5 |  |  |  |  |  |  |  |  |  |
|  | 4 | D1, D2 | 3.0 | 2.02 | 50 | 200 | 62 | 222 | 50 | 200 | 1.37 | 0.88 |  |
|  | 5 | Thumb, D1, D2 | 3.0 | 2.21 | 50 | 200 |  | 527 | 50 | 200 | 1.39 | 0.83 |  |
|  | 6 | Thumb, D1, D2 | 3.0 | 2.38 |  |  |  |  |  |  | 1.33 | 0.87 |  |
|  | 7 | Thumb, D1 | 3.0 | 1.97 | 50 | 200 |  |  |  |  |  |  |  |
|  | 8 | Thumb, D1 | 3.0 | 1.95 | 50 | 200 | 27 | 647 | 50 | 200 | 1.49 | 0.92 |  |
|  | 9 | Palm, Thumb, D1–D3 | 3.0 | 2.14 | 50 | 200 | 59 | 516 | 50 | 200 | 1.32 | 0.91 |  |
|  | 10 | D2, D3 | 3.0 | 1.98 | 50 | 200 | 44 | 488 | 50 | 200 | 1.29 | 0.86 |  |
|  | 11 | Thumb, D1, D2 | 3.0 | 2.13 | 50 | 200 | 54 | 300 | 100 | 200 | 1.32 | 0.91 |  |
|  | 12 | Hand | 3.0 |  |  |  |  |  |  |  |  |  |  |
|  | 13 | Hand | 3.0 | 1.5 |  |  |  |  |  |  |  |  |  |
|  | 14 | Hand | 2.0 | 1.95 | 50 | 200 |  |  |  |  |  |  |  |
|  | 15 | Hand | 3.0 | 2.1 | 50 | 200 |  |  |  |  |  |  |  |
|  | 16 | Thumb, D1 | 3.0 | 1.83 | 50 | 200 | 96 | 360 | 50 | 200 | 1.36 | 0.89 |  |
